# 24S-hydroxycholesterol and 25-hydroxycholesterol differentially impact hippocampal neuronal survival following oxygen-glucose deprivation

**DOI:** 10.1371/journal.pone.0174416

**Published:** 2017-03-27

**Authors:** Min-Yu Sun, Amanda Taylor, Charles F. Zorumski, Steven Mennerick

**Affiliations:** 1 Department of Psychiatry, Washington University School of Medicine, St. Louis, Missouri, United States of America; 2 Department of Neuroscience, Washington University School of Medicine, St. Louis, Missouri, United States of America; 3 Taylor Family Institute for Innovative Psychiatric Research, Washington University School of Medicine, St. Louis, Missouri, United States of America; University of Louisville, UNITED STATES

## Abstract

N-methyl-D-aspartate receptors (NMDARs), a major subtype of glutamate receptor mediating excitatory transmission throughout the CNS, participate in ischemia-induced neuronal death. Unfortunately, undesired side effects have limited the strategy of inhibiting/blocking NMDARs as therapy. Targeting endogenous positive allosteric modulators of NMDAR function may offer a strategy with fewer downsides. Here, we explored whether 24S-hydroxycholesterol (24S-HC), an endogenous positive NMDAR modulator characterized recently by our group, participates in NMDAR-mediated excitotoxicity following oxygen-glucose deprivation (OGD) in primary neuron cultures. 24S-HC is the major brain cholesterol metabolite produced exclusively in neurons near sites of glutamate transmission. By selectively potentiating NMDAR current, 24S-HC may participate in NMDAR-mediated excitotoxicity following energy failure, thus impacting recovery after stroke. In support of this hypothesis, our findings indicate that exogenous application of 24S-HC exacerbates NMDAR-dependent excitotoxicity in primary neuron culture following OGD, an ischemic-like challenge. Similarly, enhancement of endogenous 24S-HC synthesis reduced survival rate. On the other hand, reducing endogenous 24S-HC synthesis alleviated OGD-induced cell death. We found that 25-HC, another oxysterol that antagonizes 24S-HC potentiation, partially rescued OGD-mediated cell death in the presence or absence of exogenous 24S-HC application, and 25-HC exhibited NMDAR-dependent/24S-HC-dependent neuroprotection, as well as NMDAR-independent neuroprotection in rat tissue but not mouse tissue. Our findings suggest that both endogenous and exogenous 24S-HC exacerbate OGD-induced damage via NMDAR activation, while 25-HC exhibits species dependent neuroprotection through both NMDAR-dependent and independent mechanisms.

## Introduction

Ischemic stroke is a major cause of death and disability in the United States, and excitotoxicity is a major outcome of the bioenergetic failure associated with stroke [1]. Energy disruption triggers depolarization, which initiates a positive-feedback cycle of release of glutamate, an important excitatory (depolarizing) transmitter, and subsequent overactivation of N-methyl-D-aspartate receptors (NMDARs), a major subtype of glutamate receptors mediating excitatory transmission throughout the CNS. NMDARs participate in ischemia-induced neuronal injury and death by admitting Ca^2+^ and promoting excitotoxic cell death [2]. Therefore, diminishing NMDAR-mediated excitotoxicity may be neuroprotective and benefit stroke outcome. However, undesired side effects have limited the strategy of directly inhibiting/blocking NMDARs as therapy. Understanding and targeting endogenous positive allosteric modulators of NMDAR function may produce fewer downsides since basal (orthosteric) NMDAR function remains unaltered.

We have recently identified a novel class of positive NMDAR modulators– 24S-hydroxychlesterol (24S-HC) and its synthetic analogues [3]. 24S-HC is the major brain cholesterol metabolite. It is produced by cholesterol 24-hydroxylase (CYP46A1), a neuron-specific enzyme localized to dendrites [4]. 24S-HC is found abundantly in adult brain tissue (30–60 μg/g tissue), suggesting that it may regulate normal and pathophysiological NMDAR activity [5, 6]. In addition, 24S-HC has proven to be toxic through mechanisms independent of NMDARs in neuronal cell lines [7, 8].

Here we assess contributions of 24S-HC to neuronal death associated with oxygen-glucose deprivation (OGD), especially the role of NMDARs in the actions of 24S-HC. We exploit primary hippocampal neurons, where 24S-HC levels can be readily controlled and measured. We explored whether manipulating exogenous or endogenous levels of 24S-HC is neuroprotective against OGD-induced excitotoxicity. We also examined the concentrations of 24S-HC that exacerbate *in vitro* injury caused by OGD, and we explored whether exacerbation by 24S-HC can be entirely ascribed to NMDAR modulation. Moreover, we investigated whether 25-hydroxycholesterol (25-HC), an endogenous oxysterol recently shown to antagonize 24S-HC’s potentiating effects on NMDARs [9], has beneficial effects against OGD-induced neuronal death. We find that 25-HC neuroprotection is partly dependent on its antagonism of 24S-HC but surprisingly also includes a component that is independent of NMDARs. This component proved to be species dependent. In sum, targeting an endogenous positive allosteric modulator of NMDARs or perhaps targeting the novel NMDAR independent mechanism described here may avoid deleterious side effects of NMDAR antagonists as therapeutics [10].

## Materials and methods

### Cell culture

All animal care and experimental procedures were consistent with National Institutes of Health guidelines and were approved by the Washington University Animal Studies Committee. Studies involving animals are reported in accordance with the ARRIVE guidelines for reporting experiments involving animals [11, 12]. Rat or mouse hippocampal cultures were prepared from postnatal day 1 to 3, pups of both sexes (85% female) anaesthetized with isoflurane. Hippocampal slices (500 μm thickness) were digested with 1 mg·mL^−1^ papain in oxygenated LeibovitzL-15 medium (Life Technologies, Gaithersburg, MD, USA). Tissue was mechanically triturated in modified Eagle’s medium (MEM; Life Technologies) containing 5% horse serum, 5% FCS, 17 mM D glucose, 400 μM glutamine, 50 U·mL^−1^ penicillin and 50 μg·mL^−1^ streptomycin. Cells were seeded in modified Eagle’s medium at a density of ~650 cells per mm^2^ (onto 25 mm cover glasses coated with 5 mg·mL^−1^ collagen or 0.1 mg·mL^−1^ poly-D-lysine with 1 mg·mL^−1^ laminin). Cultures were incubated at 37°C in a humidified chamber with 5%CO_2_/95% air. Cytosine arabinoside (6.7 μM) was added 3–4 days after plating to inhibit glial proliferation. The following day, half of the culture medium was replaced with Neurobasal medium (Life Technologies) plus B27 supplement (Life Technologies).

### Oxygen-glucose deprivation and H_2_O_2_ toxicity

Mass cultures (13–14 DIV) were exposed to oxygen-glucose deprivation (OGD), in which original medium containing 25 mM glucose was exchanged for fresh MEM medium with 2.5 mM glucose and exposed to a sealed chamber (Billups-Rothenberg, Del Mar, CA, USA), humidified and saturated with 95% nitrogen and 5% CO_2_ at 37°C, for 2.5 h. The gas exchange followed the specifications of the chamber manufacturer (flow of 20 L·min^−1^ for 4 min to achieve 100% gas exchange). In some experiments, original medium was exchanged for fresh MEM medium containing the specified drugs immediately prior to OGD exposure. Controls were incubated in MEM medium without oxygen deprivation. Following OGD or control treatment, cells were returned to their original medium and incubated under standard culture conditions until the cell death assay (24 h later). We used Hoechst 33342 (5 μM) to identify all nuclei and propidium iodide (PI, 3 μM) for 30 min to stain nuclei of cells with compromised membranes. Mass cultures (10–14 DIV) challenged with H_2_O_2_ were incubated in 100 μM H_2_O_2_ for 60 min in the presence of 20 μM MK-801 during and after the insult to ensure no NMDAR contribution to toxicity. After a 24 h latent period cell survival was quantified as for OGD challenge.

### Chemicals

SGE-201 was synthesized by previously published methods [3] and was a gift from Sage Therapeutics. D-APV and MK-801 were purchased from Tocris (Bristol, UK). 25-HC was purchased from Sigma (St. Louis, MO).

### Measurement of 24S-HC level in cell culture medium

24-hydroxycholesterol in each medium (100 μL) sample was extracted with 400 μL of methanol. Deuterated 24-hydroxycholesterol-d_7_ (10 ng) was added to each medium sample before extraction. Extracted 24-hydroxycholesterol and the internal standard were derivatized with N,N-dimethylglycinate (DMG) to increase sensitivity. Oxysterol analysis was performed with a Shimadzu 20AD HPLC system, a LeapPAL autosampler coupled to a triple quadrupole mass spectrometer (API 4000) operated in MRM mode. The positive ion ESI mode was used for detection of derivatized oxysterols. The study samples were injected in duplicate for data averaging. Data processing was conducted with Analyst 1.5.1 (Applied Biosystems). Quantification of 24S-hydroxycholesterol was determined by the deuterium labeled internal standard spiked in each sample. 24-hydroxycholesterol data were normalized as ng/mL for various media.

### Data analysis

Five 10X microscope fields were quantified per condition per experiment, yielding >100 total neurons for each condition. Ratios of healthy neurons were quantified as the fraction of PI-negative neuronal nuclei to total neuronal nuclei. Automated cell counting algorithms (ImageJ software, National Institutes of Health, Bethesda, MD, USA) were used for cell counts. Toxicity experiments were treated as a dependent sample design [13] in which sibling cultures plated in identical media and exposed to OGD at the same time were compared by repeated measures statistics. Results are shown as means ± SEM. Comparative statistics were performed with Student’s t-test or one-way repeated measures ANOVA where indicated.

## Results

### 24S-HC exacerbates OGD damage, which is APV sensitive

**Fig 1:**
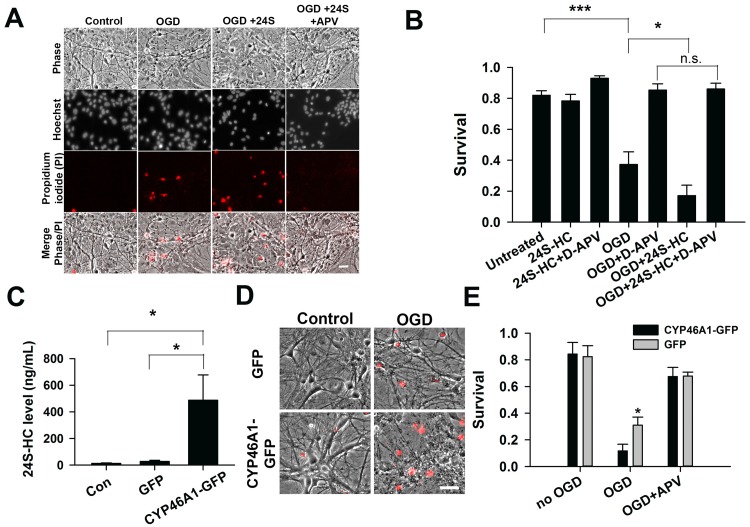
24S-HC exacerbates OGD-induced, NMDAR-dependent cell death. (A) Representative images showing propidium iodide (PI)–labeled, compromised cells in control, OGD, OGD+24S-HC (2 μM), and OGD+24S-HC+APV (50 μM). Scale bar is 30 μm. (B) Survival rate was calculated as number of dead cells normalized to total cells and compared between control, OGD, OGD+APV, OGD+24S-HC, and OGD+24S-HC+APV. Cells treated with OGD and 24S-HC exhibited significantly lower survival rate compared to those treated with OGD alone (n = 7 cultures for each group; one-way repeated measures ANOVA with Bonferroni’s post hoc test, *P < 0.05). (C) 24S-HC levels in the culture media were measured from control (no virus infection), AAV8-GFP, and AAV8-CYP46A1-GFP infected cell cultures (n = 4 cultures for each group; one-way repeated measures ANOVA and Bonferroni post hoc test, *P < 0.05). (D) Representative images showing PI-labeled dead cells in GFP-expressing cells and CYP46A1 GFP-expressing cells with or without OGD challenges. Scale bar is 30 μm. (E) Survival rate was compared between control GFP and CYP46A1 GFP-expressing cells with/without OGD, and with OGD+ APV. Following OGD treatment, CYP46A1 GFP-expressing cells had significantly lower survival rate compared to GFP-expressing cells (n = 5 cultures for each group; two-way repeated measures ANOVA with paired t test, *P <0.05).

Cell death induced by hypoxia and OGD in hippocampal cultures is NMDAR dependent [14–17]. Because 24S-HC and its analogues increase NMDAR activity [3], these compounds may exacerbate OGD-induced cell death by potentiating NMDAR activity. Effects of 24S-HC on NMDAR function saturate at ~10 μM [3]. Consistent with these observations, exogenous application of 24S-HC at 2 μM to WT rat hippocampal cultures 14 days in vitro enhanced OGD-induced cell death (Fig 1A and 1B). This cell death was rescued by co-treatment with an NMDAR antagonist, APV, prior to and during OGD, confirming that the exacerbation of cell death was NMDAR dependent [14–17].

We also tested whether elevation of 24S-HC by genetically overexpressing *CYP46A1* to mimic mature 24S-HC levels could augment endogenous 24S-HC and OGD-induced toxicity. WT rat primary hippocampal cultures were infected with an AAV-CYP46A1-GFP virus. Conditioned culture medium from cells infected with AAV-CYP46A1-GFP exhibited significant elevation of 24S-HC level compared to control AAV-GFP infected cultures (Fig 1C), verifying virus effectiveness. Overexpression of *CYP46A1* increased cell death following OGD relative to controls (Fig 1D and 1E). Again, APV prevented OGD-induced cell death in both AAV-CYP46A1-GFP and AAV-GFP-infected neuron cultures (Fig 1E), implicating NMDARs in the increased damage. Taken together, these results suggest that 24S-HC exacerbates OGD-induced damage, and its actions exclusively involve NMDAR activation. Furthermore, because OGD occurs in unconditioned culture medium devoid of 24S-HC (see Methods and below), the results suggest that locally elevated levels of 24S-HC may drive NMDAR activity to exacerbate damage.

### Endogenous 24S-HC exacerbates OGD-induced damage

**Fig 2:**
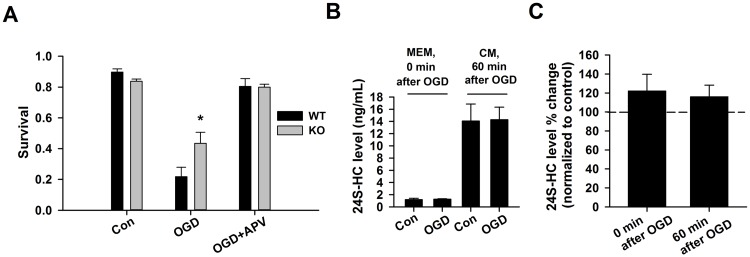
Endogenous 24S-HC exacerbates OGD-induced, NMDAR-dependent cell death. (A) Survival rate was compared between WT and KO cells with/without OGD challenges, and with OGD/APV co-treatment. Following OGD treatment, KO cells had significantly higher survival rate compared to WT cells (n = 5 cultures for WT and 5 cultures for KO; one-way ANOVA with Bonferroni’s post hoc test, *P < 0.05). (B) 24S-HC concentration (ng/ml) was measured in MEM and the original conditioned medium (CM) immediately following OGD insult and one hour after, respectively. (C) 24S-HC levels measured both immediately following OGD insult and one hour after insult, as shown in (B), were normalized to 24S-HC measured in the sibling untreated control cultures shown in (B) (n = 5 for control cultures and 6 for OGD cultures; P > 0.2).

We next investigated whether down-regulation of endogenous 24S-HC protects against OGD-induced cell death. In *CYP46A1* knockout (KO) mice, 24S-HC is greatly reduced, and deficits of NMDAR-dependent functions including basal EPSCs, synaptic plasticity, learning and memory have been reported [4, 18], suggesting reduced NMDAR activity in these mice. When comparing OGD-induced cell death in WT and KO hippocampal cultures, we observed a significantly higher survival rate in KO cultures, suggesting that reduction of endogenous 24S-HC protects against OGD-induced damage (Fig 2). This result is consistent with our previous findings in hippocampal slices, where KO slices exhibit resistance to OGD-induced synaptic depression [19].

Because basal levels of 24S-HC are expected to be low in unconditioned medium used for OGD, we were surprised at the difference in OGD neuronal survival between KO and WT cultures. We hypothesized that despite low basal concentrations, toxic insults may increase extracellular 24S-HC concentration in medium during insult to levels that modulate NMDAR dependent damage. To test whether OGD increases 24S-HC concentration to levels necessary to exacerbate OGD neuronal injury, we sampled culture medium immediately after the 2.5 h OGD insult. We found that 24S-HC concentration in OGD-challenged cultures was not different than control dishes (Fig 2B and 2C). Moreover, absolute, bulk levels of 24S-HC in the low-glucose medium of both OGD and control cultures were very low compared with 24S-HC levels in the original conditioned medium (Fig 2B), as a result of the short conditioning period of the fresh, low-glucose medium. Following insult, cells were returned to the original conditioned medium containing full glucose concentration. Levels of 24S-HC in this medium were measured 1 hr later and were not significantly elevated by the preceding OGD insult (Fig 2B and 2C). Basal levels of 24S-HC in conditioned medium from mouse cultures were 14.1 ± 2.8 ng/ml, or approximately 35 nM (Fig 2B). This concentration is likely just-threshold for eliciting NMDAR potentiation [3] but is unlikely to contribute directly to OGD injury in our conditions because conditioned medium containing 24S-HC is removed in favor of low-glucose unconditioned medium for OGD experiments (Fig 2A). Comparator 24S-HC concentrations in conditioned medium from one KO culture were 0.5 ng/ml (~1.2 nM) in baseline medium and 0.5 ng/ml (~1.3 nM) in OGD conditioned medium. Thus, the low levels in KO culture conditioned medium verify that WT 24S-HC results from ongoing enzymatic synthesis. Overall, these results suggest that bulk 24S-HC levels are unlikely to contribute to OGD damage in mouse cultures under our conditions, and local levels of 24S-HC near sites of release may mediate effects. Our results do not support the idea that 24S-HC levels are elevated by insult.

### 25-HC is partially neuroprotective against 24S-HC exacerbated damage and against damage in the absence of 24S-HC

**Fig 3:**
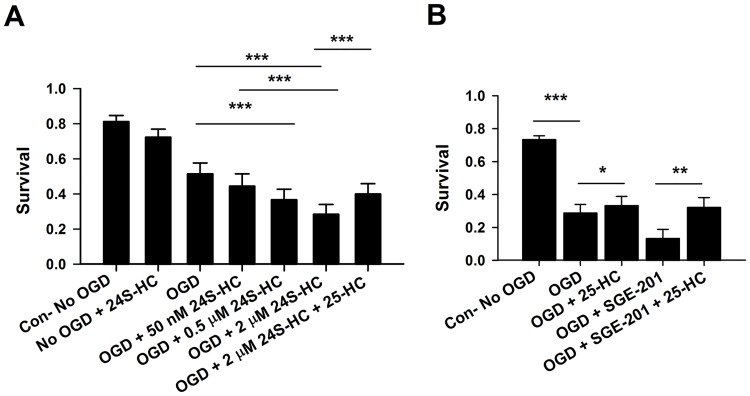
The 24S-HC exacerbation of OGD damage is concentration dependent and is partially rescued by 25-HC. (A) Survival was compared between control, OGD, OGD+50 nM 24S-HC, OGD+0.5 μM 24S-HC, OGD+2 μM 24S-HC, and OGD+2 μM 24S-HC+10 μM 25-HC. Cells treated with OGD and 24S-HC at 0.5 μM and 2 μM showed significantly poorer survival compared to those treated with OGD alone. Application of 25-HC partially prevented OGD-induced cell death exacerbated by 2 μM 24S-HC. (n = 11 cultures for each group; one-way repeated measures ANOVA with Bonferroni’s post hoc test, *P < 0.05; **P < 0.01; ***P<0.001). (B) 25-HC not only partially rescued OGD-induced cell death following SGE-201 application, but also protected against OGD-induced cell death in the absence of 24S-HC analogue application (n = 8 cultures for each group; one-way repeated measures ANOVA with Bonferroni’s post hoc test, *P < 0.05; **P < 0.01; ***P < 0.001).

OGD-induced cell death in rat cultures depended on 24S-HC concentration over a range of 50 nM to 2 μM (Fig 3A). These results suggest that the local level of 24S-HC produced in WT cells is not saturating. We recently showed that the oxysterol 25-HC non-competitively antagonizes 24S-HC effects on NMDARs [9]. Here we found that co-treatment with 10 μM 25-HC and 2 μM 24S-HC significantly alleviated OGD-induced cell death relative to that induced by 24S-HC and OGD alone (Fig 3A). Similarly, 1 μ M SGE-201, a synthetic 24S-HC analogue [3], exacerbated OGD injury, and this enhanced damage was reduced by 10 μM 25-HC (Fig 3B). Even without exogenous application of 24S-HC, OGD-induced cell death was alleviated by 10 μM 25-HC incubation during the OGD insult (Fig 3B). This result could arise from antagonism of unappreciated local, endogenous 24S-HC activity. However, because measured bulk endogenous 24S-HC levels are low, it is also possible that, in additional to antagonizing 24S-HC-induced potentiation of NMDARs, 25-HC may have NMDAR-independent neuroprotective effects against OGD damage.

### Evidence for NMDAR independent neuroprotective effects of 25-HC

**Fig 4:**
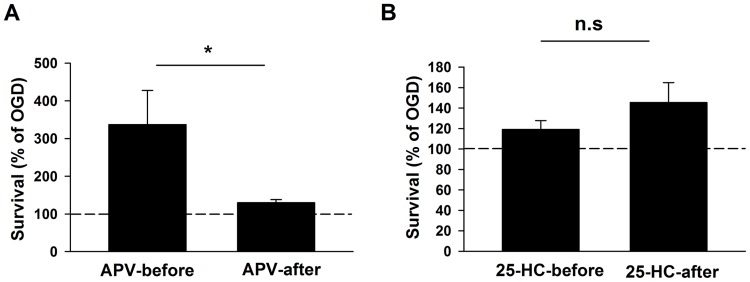
25-HC alone is neuroprotective against OGD-induced cell death, independent of NMDARs. (A) Survival rates from cultures treated with APV before or after OGD insult were normalized to the survival rate of cultures treated with OGD alone. APV (50 μM) administered before OGD insult had significantly larger neuroprotective effects on the survival rate compared to APV administered after OGD insult (n = 7 cultures for APV-before; n = 15 cultures for APV-after; *P < 0.05). (B) Survival rates from cultures treated with 25-HC before or after OGD insult were normalized to the survival rate of cultures treated with OGD alone. Administration of 25-HC (10 μM) before and after OGD insult had indistinguishable rescuing effects on survival (n = 15 cultures for each group; P > 0.2).

To test whether the entirety of 25-HC’s protective effect involves NMDARs, we applied 25-HC at different time points relative to the OGD insult, where the contribution of NMDARs is expected to vary. As a probe of NMDAR involvement at various stages during and after OGD, we tested APV neuroprotection in a separate group of cultures. As expected, APV was highly neuroprotective when administered before and during OGD. APV was much less effective when administered during the latent period following OGD insult, demonstrating a minor contribution of NMDAR activation in this period (Fig 4A). If 25-HC acts through NMDARs, we would expect a corresponding decrease in effectiveness when 25-HC is administered following insult. By contrast, cultures treated with 25-HC before or after OGD insult exhibited comparable survival rates (Fig 4B). Thus, despite the minor contribution of NMDARs following OGD, 25-HC neuroprotection was not altered, suggesting the possibility that OGD elicits a degree of NMDAR independent cell death, against which 25-HC is particularly neuroprotective.

**Fig 5:**
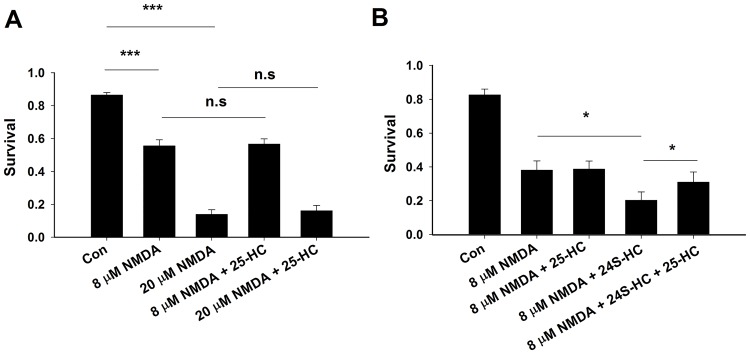
25-HC alone does not protect against NMDA-induced cell death, but it alleviates 24S-HC exacerbated toxicity. (A) Application of NMDA at 8 μM and 20 μM significantly reduced cell survival, which was not inhibited when cells were co-treated with 10 μM 25-HC (n = 13 cultures for each group, one-way repeated measures ANOVA with Bonferroni’s post hoc test, ***P < 0.001). (B) Application of 24S-HC significantly exacerbated 8 μM NMDA-induced cell death. The exacerbation was inhibited by 10 μM 25-HC (n = 6 cultures for each group, one-way repeated measures ANOVA with Bonferroni’s post hoc test, *P < 0.05).

If the neuroprotective effects of 25-HC are entirely through antagonism of NMDAR function and endogenous, local 24S-HC, we would expect that 25-HC should also reduce direct NMDA-induced cell death. To test this, NMDA was exogenously applied at either 8 μM or 20 μM to induce mild or severe neuronal injury (Fig 5A). Under these conditions, 25-HC exhibited no neuroprotection, regardless of the severity of NMDA toxicity (Fig 5A). However, co-treatment with 24S-HC exacerbated NMDA toxicity, and this exacerbation was significantly alleviated by 25-HC (Fig 5B). These results suggest that 25-HC does not affect basal NMDAR activity by antagonizing local 24S-HC actions during excitotoxicity.

**Fig 6:**
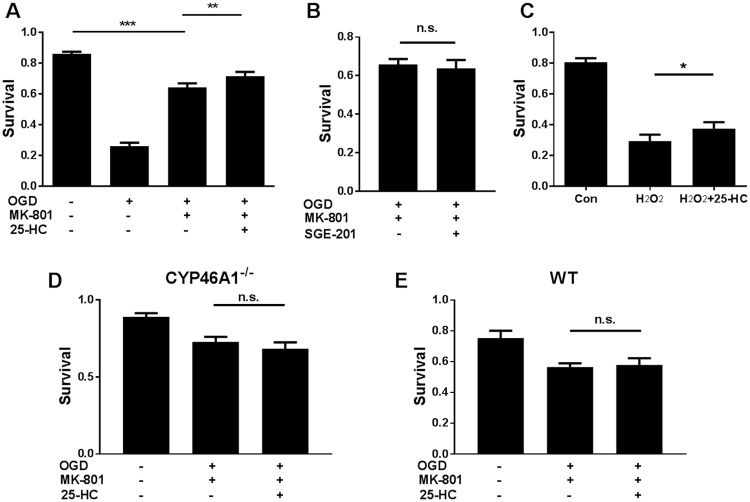
25-HC has a species-dependent, NMDAR-independent protecting effect on OGD-induced cell death. (A) Survival rate in rat cultures was compared between control, OGD (3 hr), OGD (3 hr) + MK-801(20 μM), and OGD (3 hr) + MK-801(20 μM)+ 25-HC (20 μM). Cell death induced by more severe OGD (3 hr) was only partially inhibited by MK-801 application. 25-HC protected against the MK-801 insensitive OGD-induced cell death. (n = 12 cultures for each group, one-way repeated measures ANOVA with Sidak’s post hoc test, **P < 0.01, ***P < 0.001). As in other experiments with 25-HC, neuroprotection was modest but reliable across experiments. (B) SGE-201 did not increase cell death, verifying the NMDAR independence (N = 9 cultures for each group; P > 0.05). (C) H_2_O_2_ (100 μM, 1 hour treatment) toxicity was used to test neuroprotection of 25-HC. 25-HC again yielded mild but reliable protection (N = 10 cultures for each group, one-way repeated measures ANOVA with Bonferroni’s post hoc test, *P < 0.05). (D) and (E) Neither WT nor CYP46A1 KO hippocampal neuron cultures from C57Bl/6 mice was sensitive to the mild NMDAR independent neuroprotective effect of 25-HC (N = 7 cultures for each group; P > 0.05).

Our results so far suggest that 25-HC may protect neurons during and after OGD, independent of NMDAR activity. As a final test, we applied a stronger OGD insult by increasing OGD exposure time from 2.5 h to 3–4 h to help emphasize any NMDAR-independent mechanisms of OGD-induced death. Under these conditions, cell death was only be partially rescued by NMDAR blockade with 20 μM MK-801, a non-competitive NMDAR channel blocker. To ensure that NMDARs were fully blocked under this condition, we co-treated cultures with 1 μM SGE-201. If unblocked NMDARs contribute to the residual death in the presence of MK-801, we would expect exacerbation of cell loss. In contrast to this expectation, 1 μM SGE-201 failed to exacerbate OGD toxicity in the presence of 20 μM MK-801 (Fig 6B; n = 9 cultures, P > 0.05), suggesting complete inhibition of NMDAR activity. Nevertheless, 20 μM 25-HC yielded mild but significant neuroprotection in the presence of 20 μM MK-801 (Fig 6A). This finding strongly supports the hypothesis that 25-HC is mildly neuroprotective against OGD-induced cell death through a mechanism independent of NMDAR inhibition.

To further test the NMDAR-independent neuroprotective effect, we used a model of oxidative damage: H_2_O_2_ induced toxicity. In the presence of MK-801 to eliminate contributions of NMDARs, 25-HC had a similarly mild but consistent neuroprotective effect to that observed in OGD insult (Fig 6C). Despite these consistent effects in rat cultures, we were surprised to find that the NMDAR-independent neuroprotective effect did not extend to either WT or CYP46A1^-/-^ cultures (Fig 6D and 6E). Therefore, the mild NMDAR independent neuroprotective effect of 25-HC appears to be species specific.

## Discussion

Our results extend the understanding of a class of NMDAR positive allosteric modulators that has been hiding in plain sight for decades. 24S-HC is the main cholesterol metabolite in brain and is responsible for brain cholesterol elimination as new cholesterol is synthesized [4]. Recent discoveries have demonstrated additional roles for 24S-HC, including acute modulatory effects on NMDARs [3], transcriptional effects through the liver X receptor [20] and perhaps other signaling roles as well [21, 22]. It is also a candidate biomarker for a number of neurodegenerative diseases [22–26]. The results of the present study support the idea that 24S-HC may contribute to neuronal death in certain circumstances, in addition to serving as a biomarker of cell death. Our results support the idea that in OGD, 24S-HC’s exacerbation of damage is mainly through NMDARs. By contrast a less abundant oxysterol, 25-HC, acts as a neuroprotective agent through both NMDAR-dependent and NMDAR-independent mechanisms.

Our results revealed that OGD-induced cell death exacerbated by 24S-HC can be prevented by the NMDAR antagonists APV (Fig 1B) and MK-801 (Fig 6), suggesting that 24S-HC exacerbated injury is NMDAR dependent. This is consistent with previous findings that 24S-HC specifically potentiates NMDAR function [3]. Other studies, however, also found that 24S-HC may promote toxicity that is independent of NMDARs. At concentrations above 10 μM, 24S-HC induced cell death in SH-SY5Y human neuroblastoma cells, as well as cultured cortical neurons [7]. 24S-HC can also induce apoptosis in human T-lymphoma Jurkat cells via activation of caspase 8, a mechanism not observed in SH-SY5Y cells [8]. These findings demonstrate that 24S-HC alone can induce cell death in an NMDAR-independent manner. Nevertheless, we failed to find consistent effects of 24S-HC when administered alone (Fig 1B), and the primary effect of 24S-HC in the present work was an NMDAR dependent exacerbation of OGD-induced neuronal loss. Potential explanations for differences between the above studies and ours include cell-type differences and higher 24S-HC concentrations in previous studies (10–50 μM).

Our studies included evaluation of the potential role of endogenous 24S-HC. Endogenous 24S-HC exacerbated OGD-induced cell death (Fig 2). However, because OGD was performed in 24S-HC-free medium (Fig 2B), we posit that local 24S-HC levels, near sites of release, must be responsible for the effects of genetic overexpression and underexpression of *CYP46A1*. Local 24S-HC must exceed ~50 nM (Fig 3A) [3], the exogenous concentration needed to potentiate NMDAR function. Another possibility is that 24S-HC, which is difficult to remove from cell membranes [3], was retained from conditioned medium and contributed to NMDAR damage during OGD.

Endogenous 24S-HC may be elevated by excitatory synaptic transmission [27–29]. Hippocampal cultures exhibit age-related, activity-dependent cholesterol loss that involves reactive oxygen species, CYP46A1 mobilization [27, 28], and elevated 24S-HC in culture medium [29]. In contrast, our results revealed no evidence that OGD increases 24S-HC release (Fig 2C). Nevertheless, we cannot exclude the possibility of local increases in 24S-HC concentration following OGD-induced NMDAR stimulation that were not reflected in bulk medium.

Interestingly, our results revealed neuroprotection by 25-HC against OGD-induced cell death in rat cultures, both in the presence and absence of exogenous 24S-HC application. It is unlikely that endogenous 25-HC participated in the observed neuroprotective effects since its brain concentrations are very low relative to 24S-HC and to neuroprotective concentrations of 25-HC used in our studies [6, 30]. Neuroprotection against exogenous 24S-HC exacerbated toxicity is NMDAR dependent (Figs 3 and 5B) and is consistent with our previous work demonstrating that 25-HC antagonizes 24S-HC-mediated NMDAR potentiation [9]. However, 25-HC exhibited another, small protective effect in rat tissue, revealed in the absence of 24S-HC and in the absence of NMDAR contributions (Figs 4–6). The lack of detectable effect of 25-HC on the endogenous 24S-HC actions in OGD and NMDA toxicity (Figs 4B and 5) could result from the low levels of 24S-HC present, combined with the relatively weak antagonism of 25-HC [9]. Interaction between 25-HC and oxysterol-binding proteins [31] drives cell survival in non-neuronal, proliferating cell types [32, 33]. Future work can test the relevance of this mechanism and others to OGD neuroprotection, although the importance of the NMDAR independent mechanism of 25-HC neuroprotection ultimately may be limited by species dependence (Fig 6).

## Conclusions

Results from this study support the hypothesis that 24S-HC exacerbates NMDAR-dependent excitotoxicity induced by OGD, an ischemia-like challenge. In addition, 25-HC protects against OGD-induced cell death, even when administered following the insult. While 25-HC rescues NMDAR-dependent cell death exacerbated by 24S-HC, 25-HC exhibits species-dependent, NMDAR-independent neuroprotection against OGD-induced cell death. Our findings suggest that two oxysterols may both contribute to severity of damage and be palliative targets in ischemic stroke.

## Supporting information

S1 Dataset S1_All figures. This excel file contains the dataset used for calculating the means, standard deviations and standard errors for each figure.
(XLSX)

S2 Dataset S2_Minimal data set. This pdf file contains the values used to build graphs for each figure.
(PDF)
