# Supplementary material for: 24S-hydroxycholesterol and 25-hydroxycholesterol differentially impact hippocampal neuronal survival following oxygen-glucose deprivation
Source: PLoS One. 2017 Mar 27;12(3):e0174416. doi: 10.1371/journal.pone.0174416 (PMC5367825; doi:10.1371/journal.pone.0174416)
Supplement: S2 Dataset — This pdf file contains the values used to build graphs for each figure. (PDF) [file pone.0174416.s002.pdf]

What data are required and what is meant by minimal data set? PLOS defines the “minimal data set” to consist of the data set used to reach the conclusions drawn in the manuscript with related metadata and methods, and any additional data required to replicate the reported study findings in their entirety. Authors do not need to submit their entire data set, or the raw data collected during an investigation. Please submit the following data:

- The values behind the means, standard deviations and other measures reported;
- The values used to build graphs;
- The points extracted from images for analysis.

Fig. #	Mean	S.D	S.E	Statistical method used	P value	# samples
**Fig. 1B**				One way repeated measures ANOVA/Bonferroni: compare selected pairs	*P <0.05<br>***P <0.0001	
Untreated	0.8218	0.0768	0.029			7
24S-HC	0.784	0.1107	0.0418			7
24S-HC + D-APV	0.9305	0.0431	0.0163			7
OGD	0.373	0.2153	0.0814			7
OGD+D-APV	0.8538	0.1072	0.0405			7
OGD+24S-HC	0.1719	0.1782	0.0674			7
OGD+24S-HC+D-APV	0.8604	0.0993	0.0375			7
**Fig. 1C**				One way repeated measures ANOVA/Bonferroni: compare selected pairs	*P <0.05	
Con	12.9588	6.602	3.301			4
GFP	26.5838	17.859	8.9296			4
CYP46A1-GFP	486.5	381.2	190.6			4
**Fig. 1E**				Two way repeated measures ANOVA/paired t test	*P <0.05	
No OGD-CYP46A1-GFP	0.8429	0.1951	0.0872			5
No OGD-GFP	0.8238	0.1824	0.0816			5
OGD-CYP46A1-GFP	0.1171	0.1113	0.0498			5
OGD-GFP	0.3102	0.1350	0.0604			5
OGD+APV-CYP46A1-GFP	0.6745	0.1524	0.0681			5
OGD+APV-GFP	0.678	0.0649	0.0290			5
**Fig. 2A**				Two way ANOVA/ Bonferroni: compare selected pairs	*P <0.05	
Con_WT	0.8514	0.1082	0.0484			5
Con_KO	0.8374	0.0321	0.0143			5
OGD_WT	0.1863	0.1266	0.0566			5
OGD_KO	0.4345	0.1606	0.0718			5
OGD+APV_WT	0.7077	0.2059	0.1029			4
OGD+APV_KO	0.7999	0.0427	0.0191			5
**Fig. 2B**				Independent T test	P > 0.1	
MEM_Con	1.18	0.506	0.2263			5
MEM_OGD	1.25	0.1652	0.0675			6
CM_Con	14.06	6.2653	2.8019			5
CM_OGD	14.30	5.0237	2.0509			6
**Fig. 2C**				Paired T test	P > 0.1	
0 min after OGD	121.78	43.27	17.67			6
60 min after OGD	115.51	30.15	12.31			6

Fig. #	Mean	S.D	S.E	Statistical method used	P value	# samples
**Fig. 3A**				One way repeated measures ANOVA/Bonferroni: compare selected pairs	*P < 0.05<br>**P < 0.01<br>***P < 0.001	
Con-No OGD	0.8127	0.1146	0.0346			11
No-OGD+24S-HC	0.7239	0.1525	0.046			11
OGD	0.5151	0.2044	0.0616			11
OGD+50 nM 24S-HC	0.4447	0.2333	0.0703			11
OGD+0.5 μM 24S-HC	0.3674	0.1995	0.0602			11
OGD+2 μM 24S-HC	0.2845	0.1855	0.0559			11
OGD+2 μM 24S-HC+25-HC	0.4007	0.1938	0.0584			11
**Fig. 3B**				One way repeated measures ANOVA/Bonferroni: compare selected pairs	*P < 0.05<br>**P < 0.01<br>***P < 0.001	
Con-No OGD	0.7442	0.0645	0.0228			8
OGD	0.2515	0.1361	0.0481			8
OGD+25-HC	0.3052	0.1359	0.0480			8
OGD+SGE-201	0.1073	0.1250	0.0442			8
OGD+SGE-201+25-HC	0.2759	0.1508	0.0533			8
**Fig. 4A**				Mann-Whitney non-paired test	*P < 0.05	
APV-before	337.12	240.17	90.78			7
APV-after	129.87	31.59	8.16			15
**Fig. 4B**				Wilcoxon matched Pairs T test	P > 0.1	
25-HC-before	119.10	33.45	8.64			15
25-HC after	145.41	75.43	19.47			15
**Fig. 5A**				One way repeated measures ANOVA/Bonferroni: compare selected pairs	***P < 0.001	
Con	0.8642	0.0553	0.0153			13
8 μM NMDA	0.5553	0.1359	0.0377			13
20 μM NMDA	0.1388	0.1001	0.0278			13
8 μM NMDA + 25-HC	0.5669	0.1111	0.0308			13
20 μM NMDA + 25-HC	0.1606	0.1159	0.0321			13
**Fig. 5B**				One way repeated measures ANOVA/Bonferroni: compare selected pairs	*P < 0.05	
Con	0.8253	0.0848	0.0346			6
8 μM NMDA	0.3805	0.1353	0.0553			6
8 μM NMDA + 25-HC	0.387	0.1169	0.0477			6
8 μM NMDA + 24S-HC	0.2022	0.1207	0.0493			6
8 μM NMDA + 24S-HC + 25-HC	0.31	0.1480	0.0604			6
**Fig. 6A**				One way repeated measures ANOVA/Sidak's test: compare selected pairs	**P < 0.01<br>***P < 0.001	
Con	0.8588	0.0506	0.0146			12
OGD	0.260	0.0798	0.0230			12
OGD+MK-801	0.6408	0.0970	0.0280			12
OGD+MK-801+25-HC	0.7128	0.1033	0.0298			12
**Fig. 6B**				Paired two-tailed T test	P > 0.05	
OGD+MK-801	0.6573	0.0876	0.0292			9
OGD+MK-801+SGE-201	0.6372	0.1324	0.0441			9

Fig. #	Mean	S.D	S.E	Statistical method used	P value	# samples
**Fig. 6C**				One way repeated measures ANOVA/Bonferroni: compare selected pairs	*P < 0.05	
Con	0.8058	0.0846	0.0268			10
H_2O_2	0.2922	0.1372	0.0434			10
H_2O_2+25-HC	0.3746	0.1311	0.0415			10
**Fig. 6D**				One way repeated measures ANOVA/Dunnett's test: compare selected pairs	P > 0.05	
Con (CYP46A1-/-)	0.8882	0.0648	0.0245			7
OGD+MK-801(CYP46A1-/-)	0.7272	0.0858	0.0324			7
OGD+MK-801+25-HC (CYP46A1-/-)	0.6821	0.1108	0.0419			7
**Fig. 6E**				One way repeated measures ANOVA/Dunnett's test: compare selected pairs	P > 0.05	
Con (WT)	0.7532	0.1270	0.0480			7
OGD+MK-801 (WT)	0.5654	0.0639	0.0242			7
OGD+MK-801+25-HC (WT)	0.5793	0.1146	0.0433			7
